# The thickness-induced magneto-transport and optic properties enhancement in Sb_2_Te_3_ flakes

**DOI:** 10.1038/s41598-018-34857-y

**Published:** 2018-11-12

**Authors:** Shiu-Ming Huang, Kai-Jui Chen, You-Jhih Yan, Shih-Hsun Yu, Mitch Chou

**Affiliations:** 1 Department of Physics, National Sun Yat-Sen University, Kaohsiung, 80424 Taiwan; 2 Department of Materials and Optoelectronic Science, National Sun Yat-Sen University, Kaohsiung, 80424 Taiwan; 3 Taiwan Consortium of Emergent Crystalline Materials, TCECM, National Sun Yat-Sen University, Kaohsiung, 80424, Taiwan

## Abstract

The electric and optical properties were studied in Sb_2_Te_3_ with different thickness. It reveals the same resistivity at measured temperatures, but shows a larger magnetoresistance ratio at thicker flakes. All measured data conformed to a linear correlation between magnetoresistance ratio which is one-order enhanced, and mobility over a wide mobility range. A higher photocurrent response is observed in thicker flakes. These results support that the thickness enhances the effective carrier mobility which leads to magneto-transport and optic properties enhancement.

## Introduction

Topological insulators (TIs) are characterized by their distinctive surface states with linear dispersion^1–9^. The effective carrier characteristic is a combination of carriers from the surface state and the bulk state. To optimize the transport and optic characteristics, the Fermi level tuning through back-gate voltage^10,11^, as well as material component adjustment^11–16^ are widely investigated.

It is widely known that the carrier transport is tolerant to the extrinsic structure defeats and non-magnetic impurities in TIs, thus the oxidation and non-magnetic molecular pollution should reveal weak effect on carrier transport properties^1,2^. However, previous studies have shown that, carrier transport characteristics are extremely spread over a wide range. It reveals that the magnetoresistance (MR) and the residual resistance ratio follow the same tendency over a wide range of thickness. This inspires us that other than the sample quality, the geometric size might be a critical factor on these transport characteristics^17–20^.

In this work, we perform the MR in the Sb_2_Te_3_ TI with different thicknesses, which are exfoliated from the same crystal. It shows the same temperature dependent resistivity in all flakes, and that indicates high uniformity and quality in our Sb_2_Te_3_ TI. It reveals larger MR ratios in thicker flakes. The mobility is a physical characteristic that shows how quickly a carrier could move through a system under an applied electric field. It is widely proposed that the carrier mobility dominates the non-saturating MR. Our analysis shows that the MR ratio is proportional to the effective carrier mobility and all measured MR ratio collapses onto a single line. These results support that the enhanced MR ratio originates from the enhanced carrier mobility that is induced by increasing flake thickness. Furthermore, a higher photocurrent response is observed in thicker flakes. These observations show that one might greatly improve and optimize the carrier transport characteristics through the thickness treatment.

## Experimental Methods

**Figure 1:**
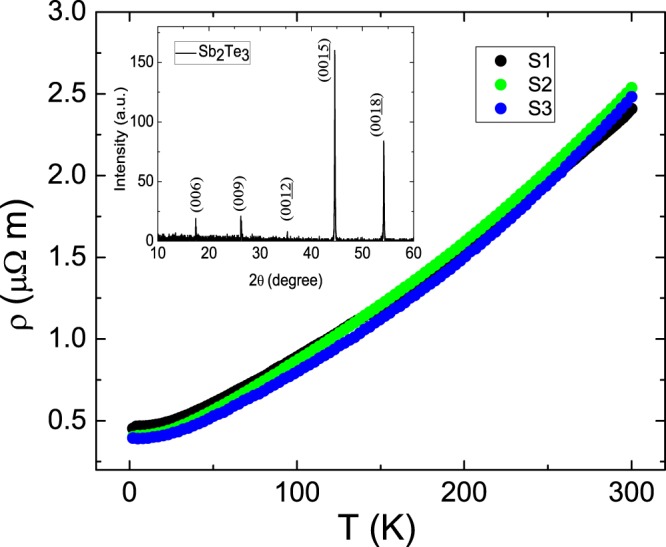
The temperature dependent resistivity of three flakes with different thicknesses. These flakes show the same resistivity and temperature dependence from 300 K to 4 K. The residual resistance ratio is 0.18 ± 0.02. The inset shows the XRD spectrum of the crystal and it is consistent with the Sb_2_Te_3_ database. These sharp diffraction peaks support the Sb_2_Te_3_ is highly crystalline.

Single crystals of Sb_2_Te_3_ were grown using a homemade resistance-heated floating zone furnace (RHFZ). The raw materials used to make the Sb_2_Te_3_ crystals were mixed according to the stoichiometric ratio. At first, the stoichiometric mixtures of high purity elements Sb (99.995%) and Te (99.995%) was melted at 700~800 °C for 20 hours and slowly cooled to room temperature in an evacuated quartz tube. The resultant material was then used as a feeding rod for the RHFZ experiment. Our previous work demonstrated that TI with extremely high uniformity can be obtained using the RHFZ method^8,9,21–23^. After growth, the crystals were furnace cooled to room temperature. The as-grown crystals were cleaved along the basal plane, using a silvery reflective surface, and then prepared for the further experiments. Energy-dispersive X-ray spectroscopy (EDS) confirmed that the crystals contain Sb:Te = 2:3. The inset in Fig. 1 shows the XRD spectrum of the crystal and it is consistent with the Sb_2_Te_3_ database. These sharp diffraction peaks support the Sb_2_Te_3_ is highly crystalline.

To identity the influence of the thickness on the intrinsic transport characteristics and optimize the factor of thickness on the mobility, three flakes with different thicknesses were prepared for transport measurements. These Sb_2_Te_3_ flakes were cleaved from the same one single crystal using the Scotch-tape method. The thickness of three flakes are 9, 14.7 and 254 *μ*m, respectively. The surface geometry was approximately 6-mm × 5-mm. Gold wires were electrically attached to the cleaved crystal surface using a silver paste. Magnetotransport measurements were performed using the standard six-probe technique in a commercial apparatus (Quantum Design PPMS) with a magnetic field of up to 9 T. The magnetic field was applied perpendicular to the large cleaved surface.

## Results and Discussion

Figure 1 shows temperature dependent resistivity of three Sb_2_Te_3_ with different thicknesses. It reveals the same resistivity and temperature dependence over a wide range of temperatures. The room temperature resistivity is roughly 2.5 ± 0.1 *μ*Ω-m for three flakes. Residual resistance at low temperature is strongly related to structure defects and impurities. The residual resistance ratio (RRR), which is defined as *R*(4K)/*R*(300K) in this work, is a factor indicating the crystal quality. The RRR is 0.18 ± 0.02 for three flakes, and it is consistent with reported values in the Sb_2_Te_3_ under different growing methods and conditions^18^. These results indicate the excellent uniformity and high quality of our Sb_2_Te_3_ crystal. Based on these observations, one could rule out the extrinsic pollution that might penetrate into our system during the flake preparation and transfer procedure. The observed transport behavior would mainly originate from intrinsic carrier properties.

**Figure 2:**
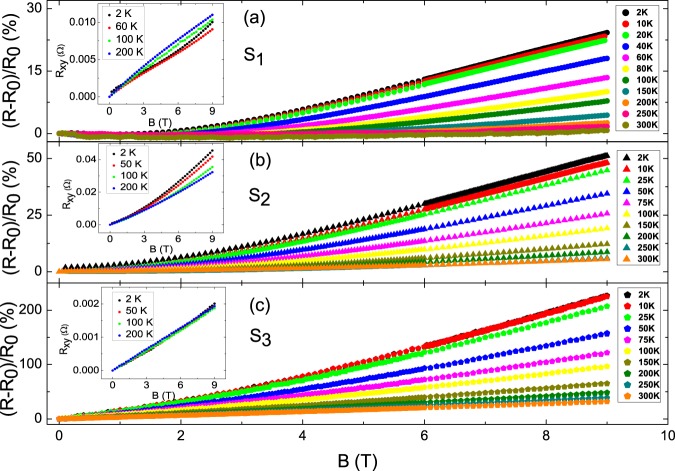
The MR ratio as a function of magnetic fields from room temperature to 2 K. It shows that the MR ratio is different in flakes with different thicknesses. The larger MR ratio is observed in thicker flakes and at lower temperatures. The insets show the Hall resistances. The Hall resistances are not linear with magnetic field due to two-carrier sources.

Figure 2 illustrates the MR ratio, (*R*(*B*) − *R*(*B* = 0))/*R*(*B* = 0), of three newly cleaved flakes with different thicknesses. Non-saturating MR was observed at magnetic fields of up to 9 T over a wide temperature ranges. It reveals larger MR ratio in thicker flakes. The MR ratios at 2 K and 9 T are 25%, 50% and 220% for flakes with the thickness of 9, 14.7 and 254 *μ*m, respectively, and found a larger MR ratio in a thicker sample. The previous works reveal different MR ratios in various kinds of TIs with different thicknesses, and it reveals a larger MR ratio in a thicker sample^17–20^. This behavior is consistent with our observation, but we found that samples with different thicknesses reveal different RRR and the temperature dependence are completely different in all previous reports. It reveals that samples with larger MR ratios reveal larger RRR in all previous reports^17–20^. The carrier transport properties are extremely sensitive to the crystal quality (RRR). Other than the thickness effect, the observed higher MR ratio in previous reports might originate from the quality effect in different samples^17–20^. In contrast with previous studies^17–20^, the sample quality are extremely controlled and reveals high uniformity. One can rule out the crystal quality and structure defect effects in our observed different MR ratios. The insets show the Hall resistances as a function of magnetic fields, and it is not linear with magnetic fields due to the two-carrier source. The relation could be expressed as:

$${R}_{xy}=-\,(\frac{B}{e})\frac{({n}_{1}{\mu }_{1}^{2}+{n}_{2}{\mu }_{2}^{2})+({n}_{1}+{n}_{2}){\mu }_{1}^{2}{\mu }_{2}^{2}{B}^{2}}{{(|{n}_{1}|{\mu }_{1}+|{n}_{2}|{\mu }_{2})}^{2}+{({n}_{1}+{n}_{2})}^{2}{\mu }_{1}^{2}{\mu }_{2}^{2}{B}^{2}}.$$

The *n*_1_ and *n*_2_ are the carrier concentrations of the surface and the bulk states, respectively. The *μ*_1_ and *μ*_2_ are the carrier mobility of the surface and the bulk states, respectively. The ${R}_{xy}\approx -\,\frac{B}{e({n}_{1}+{n}_{2})}\propto B$ at high magnetic fields^24^. To obtain the effective carrier mobility, we extracted the effective carrier concentration through fitting Hall resistances at high magnetic fields. The carrier concentration ranges from 6 × 10^25^ to 5 × 10^26^ (m^3^), which is consistent with reported values.

**Figure 3:**
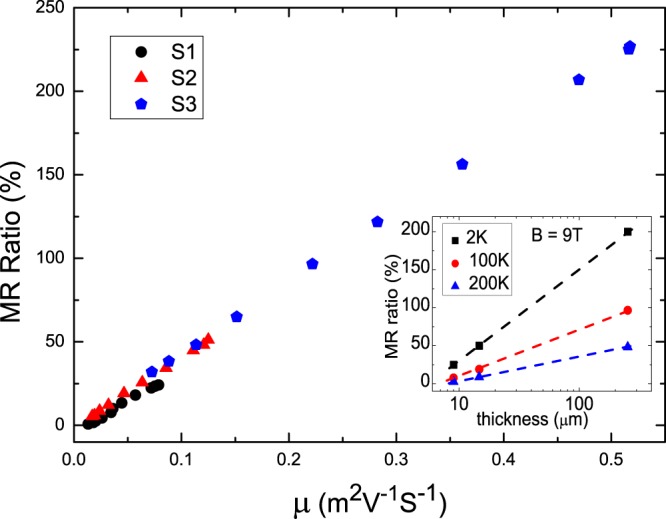
The MR ratio as a function of effective carrier mobility. The MR ratio ranges from 0.74% to 225%, and the mobility ranges from 0.004 to 0.55. The MR ratio and mobility spread over 2 orders. It reveals that the MR ratio is proportional to the effective carrier mobility. The inset shows the MR ratio as a function of the flake thickness. It reveals that the MR ratio is approximately proportional to the thickness in the logarithm plot.

Figure 3 shows the MR ratio as a function of the mobility. It reveals that all data points collapse onto a single line. The MR ratio is proportional to the carrier mobility. This supports the observed MR ratio enhancement originates from the carrier mobility enhancement. The inset of Fig. 3 reveals that the MR ratio is approximately proportional to the thickness in the logarithm plot. It comes to our attention that the reported MR ratio is about 10% for Sb_2_Te_3_ thin films with a thickness of 30 nm. This result is consistent with our presumption, even though the sample fabrication conditions and quality are different. Herewith, we would like to emphasize that the MR ratio would not continually increase with thickness without restriction. We believe that this thickness enhanced mobility effect would terminate at a characteristic thickness that is related to some intrinsic carrier transport characteristics. To the best of our knowledge, the related studies are lacking and it needs further experimental and theoretical investigations to clarify this mechanism.

**Figure 4:**
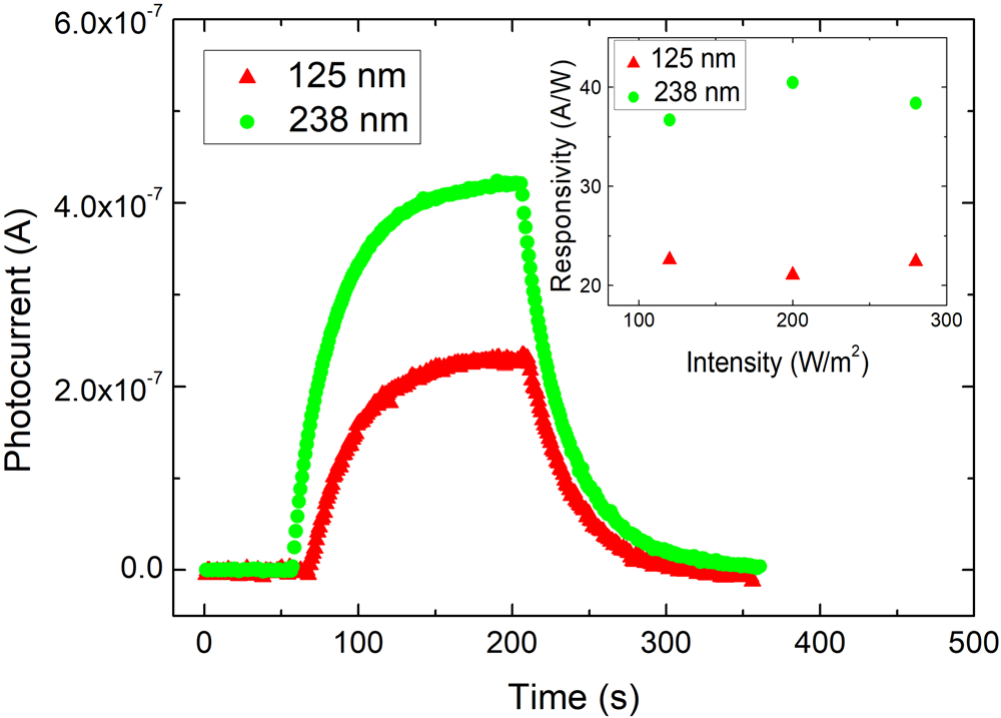
The photocurrent as a function of time in flakes with different thicknesses. The photocurrent is larger in the thicker flake. The inset shows the responsivity as a function of light power intensity in flakes with different thickness. The responsivity is independent of the power intensity. The responsivity is larger in the thicker flake.

The photocurrent response weakly depends on sample thicknesses in various kinds of materials in previous reports when the light penetration depth is shorter than the flake thickness. To confirm the observed MR ratio enhancement is originated from the intrinsic carrier mobility enhancement, the photocurrent is performed on flakes with different thickness. The light penetration length is roughly 10 nm in topological materials and that is shorter than the thickness in our experiments^25^. As shown in the Fig. 4, a larger photocurrent is observed in thicker flakes. The photocurrent is related to the surface area and light intensity. To exclude these extrinsic effects on measured photocurrents and quantitatively determine the performance under illumination, the responsivity, *R*_*es*_, is calculated through the following equation;

1 $${R}_{es}=\frac{{I}_{P}}{PS}=\frac{V\mu }{PS{l}^{2}}\propto \mu ,$$

where *I*_*P*_, *P*, *S*, *V*, *μ* and *l* are the photocurrent, the light intensity, the effective area, the applied voltage, the mobility and the electrode distance, respectively. The *R*_*es*_ is proportional to the mobility, and independent of the surface area and light intensity. Figure 4 inset shows *R*_*es*_ as a function of light power intensity. The *R*_*es*_ is larger in the thicker flake and independent of the light power intensity. This supports that thicker flakes exhibit higher carrier transport characteristics and photo response. On the other hand, the light penetration length is related to the light power intensity. As shown in the inset of Fig. 4, the *R*_*es*_ is independent of the power intensity in both flakes. This further implies that the light penetration length should be shorter than the flake thickness. Also, the effect of different carrier contribution ratios, which might come from the different light penetration length, is weak in our observation. These findings support that the observed *R*_*es*_ enhancement comes from the intrinsic carrier mobility enhancement, and the effective carrier mobility is enhanced in thicker flakes.

**Figure 5:**
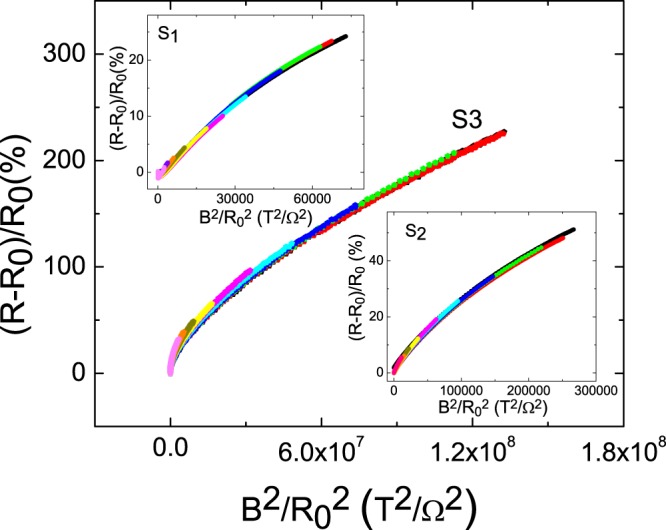
The Kohler plot of three flakes. It collapses onto a single curve in each flake.

Recently, it is reported that the effective carrier mobility is extremely enhanced in a system with physical adsorbed moleculars on the flake surface^26^. All flakes are prepared under the same processes and conditions in our experiment. Furthermore, this surface adsorption effect is mainly on the surface and should reveal weak thickness dependence. Thus, this effect should be not be the main mechanism on our observed carrier mobility enhancement. This enhancement leads to that the carrier transport properties deviating from intrinsic transport behaviors, and the measured MR would not collapse onto a single curve in the Kohler plot. The Fig. 5 shows the Kohler plot of three flakes with different thicknesses. All measured data collapse onto single curve in each flakes but exhibits different dependence. Furthermore, these macroflakes are prepared from the same one crystal with high uniformity. Different molecular adsorption level would lead to different residual resistances, and that is inconsistent with our observations. The reported molecular physical adsorption effect should be equivalent in three flakes so it is not the dominant effect in our observation.

The question arises to that what is the mechanism of the carrier mobility enhancement in thicker flakes. It is theoretically proposed that the mobility of conventional thin films depends on the film thickness as *μ*(*t*) = *μ*_∞_/(1 + 2(*λ*/*t*)(1 − *p*)), where *μ*_∞_ is the mobility of the film when the thickness, *t*, is much larger than than the mean free path, *λ*. *p* is the fraction of carriers reflecting specularly from the surface. This model describes the case in which the thickness is smaller than the mean free path, and will be independent with the thickness when thickness is much larger than the mean free path. The *λ* of our Sb_2_Te_3_ is estimated to be of order nm that is much smaller than our flake thickness^20^. As shown in the Fig. 3, our experimental result shows that the mobility increases as the thickness increases and no obvious saturation tendency. It is worthy to pay attention to that several reported works reveal that MR ratios show no saturation tendency in a flake with a thickness ranging from nm to bulk^17–20^. Like in the previous reports, our work reveals that the MR ratio is higher in thicker flakes in various kinds of topological materials^17–20^. The related theoretical discussion on this effect is lacking and intrinsic mechanism is still not clear. To identify the thickness induced MR enhancement in various kinds of materials, further theoretical discussion is necessary. Our experimental reports could provide more information on the enhancement of transport and optic characteristics in topological materials through a simple geometric treatment. Moreover, this could provide more information to clarify the intrinsic mechanism of the non-saturating MR.

## Conclusion

The electric and optical properties were studied in the Sb_2_Te_3_ with different thickness. It reveals the same resistivity at measured temperatures, but shows a larger magnetoresistance ratio at thicker flakes. All measured data conformed to a linear correlation between magnetoresistance ratio and mobility over a wide mobility range. The magnetoresistance ratio is one-order enhanced. A higher photocurrent response is observed in thicker flakes. These results support the thickness enhances the effective carrier mobility which leads to magneto-transport and optical properties enhancement.
